# Intracranial pressure is elevated at 24 h post‐stroke in mice

**DOI:** 10.1002/nep3.36

**Published:** 2024-02-25

**Authors:** Kirby E. Warren, Rebecca J. Hood, Fredrick R. Walker, Neil J. Spratt

**Affiliations:** ^1^ School of Biomedical Sciences and Pharmacy University of Newcastle Callaghan Australia; ^2^ Hunter Medical Research Institute New Lambton Heights Australia; ^3^ School of Biomedicine, Discipline of Anatomy and Pathology, Faculty of Health and Medical Sciences The University of Adelaide Adelaide Australia; ^4^ Department of Neurology John Hunter Hospital New Lambton Heights Australia

**Keywords:** cerebral edema, intracranial pressure, ischemic stroke

## Abstract

**Background:**

It has long been assumed that post‐stroke intracranial pressure (ICP) elevation occurs because of large infarct and edema volumes. However, we have repeatedly shown ICP elevation at 24 h post‐stroke in the presence of little to no edema in rats. Biological processes are often conserved across species and types of injury. Therefore, we aimed to determine if an ICP rise occurs at 24 h post‐stroke in the presence of small infarct and edema volumes in mice.

**Methods:**

Mice were randomized by random number table to either photothrombotic stroke or sham surgery (*n* = 15). Epidural ICP was recorded using a fiber optic catheter at 1 h post‐stroke (baseline) and between 23 and 24 h post‐stroke.

**Results:**

ICP was significantly higher at 24 h compared to baseline in stroke animals (*n* = 6; 10.71 ± 6.45 mmHg vs. 3.74 ± 2.20 mmHg, respectively; *p* = 0.03). ICP at 24 h was also significantly higher in stroke mice compared to sham (*n* = 6; 3.45 ± 1.43 mmHg; *p* = 0.02). There was no change in ICP in sham mice (*p* = 0.9). Edema volumes in stroke animals were small (0.04 ± 0.04 mm^3^) and unlikely to have caused significant ICP elevation.

**Conclusion:**

This study provides evidence of an edema‐independent ICP elevation following small ischemic stroke in mice. The occurrence of this rise supports our findings in other species and suggests it is caused by a previously undescribed mechanism.

## Highlights

Significant findings of the study

Intracranial pressure (ICP) is significantly increased at 24 h post‐stroke in mice in an edema‐independent manner, in line with findings in rats.

What this study adds to the advances in the areas of basic, translational or clinical neuroscience

This study highlights the need to consider mechanisms other than edema in the cause and treatment of post‐stroke ICP elevation.

## Graphical abstract

This study aimed to determine if an intracranial pressure (ICP) rise occurs in mice at 24 h post‐stroke in the presence of small infarct and edema volumes. This study finds ICP is significantly increased at 24 h post‐stroke in mice in an edema‐independent manner, in line with findings in rats. The study highlights the need to consider mechanisms other than edema in the cause and treatment of post‐stroke ICP elevation. Picture is created with BioRender.com.

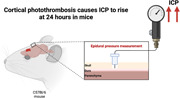

## 1 INTRODUCTION

Cerebral edema has often been the assumed cause of post‐stroke intracranial pressure (ICP) elevation in both experimental and clinical settings but has only been studied after large strokes. As a result, ICP elevation is often assumed to only be a complication of large strokes. However, there is a growing body of evidence that challenges this assumption.^1^ Experimental studies by our group^2^, ^3^, ^4^, ^5^ and others in rats^6^, ^7^ and sheep^8^ have shown that ICP is elevated 24 h post‐stroke in the presence of small infarcts. In rats we have consistently shown that a significant ICP rise also occurs despite little and, sometimes, no edema, in young^3^, ^5^, ^9^ and aged^4^ animals and using both photothrombotic and middle cerebral artery occlusion stroke models.^3^, ^4^, ^5^

The significance of this ICP rise is that we have shown increasing ICP proportionally decreases collateral blood flow after stroke.^10^ This is clinically relevant as reduced collateral supply could compromise the penumbra and cause infarct expansion and larger total infarct volume.^11^, ^12^ Having robustly characterized this ICP rise in rats, our next step is to understand the mechanisms underlying it. However, studying changes in ICP and underlying mechanisms in humans is challenging, since established ICP monitoring technology is invasive, and therefore hard to justify in those with small stroke. Noninvasive technology is in development, but not yet validated in stroke.^13^ Therefore, mechanistic investigations remain preclinical.

Our existing studies of post‐stroke ICP elevation have been performed in rats and have shown an edema‐independent mechanism of ICP rise.^3^, ^4^, ^5^, ^6^, ^9^ According to the Monro‐Kellie doctrine, mechanisms for ICP elevation other than edema are increased blood or cerebrospinal fluid (CSF) volume.^14^, ^15^ Mice offer some advantages for mechanistic studies, particularly for studying the CSF system.^16^, ^17^, ^18^, ^19^ However, there is yet no evidence for a similar ICP elevation in mice. Pathophysiological mechanisms and responses to them are often conserved across species and we hypothesized that ICP elevation, as seen in rats, could also occur in mice. Based on this, the aim of this study was to determine if mice experience an edema‐independent increase in ICP at 24 h post‐stroke.

## 2 METHODS

### 2.1 Animals

Experiments were performed on male C57BL/6 mice at the University of Newcastle (*n* = 15, 10−14 weeks, Animal Services Unit, University of Newcastle). Procedures were conducted in accordance with the Australian Code for the Care and Use of Animals for Scientific Purposes and approved by the Animal Care and Ethics Committee of the University of Newcastle (approval No.A‐2013‐340).

### 2.2 Anesthesia and monitoring

For stroke induction, mice were anesthetised with isoflurane (5% induction, 1.5−2.5% maintenance in 100% medical oxygen). Following stroke surgery, mice were switched to intraperitoneal 50−100 mg/kg ketamine and 5−10 mg/kg xylazine in 100% medical air for additional surgical procedures and physiological monitoring periods. All incision sites were subcutaneously injected with 2% bupivacaine 10 min before incision. Mean arterial pressure was monitored between 23 and 24 h post‐stroke from a right femoral artery catheter. Heart rate was monitored continuously from ICP traces. Body temperature was continuously monitored throughout surgery via a rectal probe coupled to a thermoregulatory heat mat. For recovery, animals received 1.5 mL of subcutaneous 0.9% saline to prevent dehydration and were returned to their home cage with free access to softened chow and water.

### 2.3 Photothrombotic stroke

Animals were randomized by number to stroke or sham groups before intraperitoneal injection. The photothrombotic stroke model was used, as previously described^20^ with minor modifications. Mice were given an intraperitoneal injection of 10 mg/mL (200 µL total) Rose Bengal (Sigma Aldrich, Germany) in 0.9% saline (stroke) or saline only (sham). Following 10 min of absorption, a cold light source was placed over the skull 2.2 mm lateral to Bregma, and the skull illuminated for 15 min. Mice were then prepared for ICP measurement. Animals were excluded from analysis if there was no evidence of infarct on histology.

### 2.4 ICP measurement

Epidural ICP measurement was adapted from the method described by Murtha et al. (2012).^21^ ICP measurements were obtained under anesthesia using a fiber optic catheter (Opsens, Canada). A hollow, fluid filled metal screw was inserted into the occipital bone 5 mm posterior to Bregma and the skull covered in dental cement (Vertex Dental B.V). The ICP sensor was inserted into the screw, placed above the dura and sealed in place with caulking material (Gunz Dental, Germany). ICP recordings were taken for 30 min at 1 h post‐stroke (baseline), and from 23 to 24 h post‐stroke. Cerebral perfusion pressure was measured as mean arterial pressure—ICP.

### 2.5 Histological analysis

At 24 h post‐stroke, mice were overdosed with sodium pentobarbital (200 µL, 325 mg/mL) and transcardially perfused with 0.9% saline. Brains were removed and 1 mm sections taken and incubated at 37°C in 2% 2,3,5‐Triphenyl tetrazolium chloride (TTC) for 10 min. Then, the anterior and posterior side of the section was imaged using a digital camera (HDR‐PJ790, Sony) for infarct and edema calculation. Infarcted tissue was identified as the non‐TTC‐stained region.^20^ Using ImageJ, the infarct and the ipsilateral and contralateral hemispheres were traced for calculation of edema and infarct volume:

$$\begin{array}{l} \mathrm{Edema}\;\mathrm{volume}\\ \;=\frac{ipsilateral\;hemisphere-contralateral\;hemisphere}{contralateral\;hemisphere} \end{array}$$

$$\begin{array}{l} \text{Infarct volume}\;(\text{corrected for edema})\\ \;\;=infarct\;volume\times \frac{contralateral\;volume}{ipsilateral\;volume} \end{array}\;$$

### 2.6 Statistics

Data were checked for normality using the Shapiro‐Wilk test. Statistical analysis was performed using GraphPad Prism software (version 8). Unpaired *t*‐tests were used to compare differences between stroke and sham and paired *t*‐tests were used to detect changes from baseline within group. Significant differences were accepted at *p* < 0.05. Data is presented as mean ± standard deviation (SD).

## 3 RESULTS

Three mice were excluded due to death (*n* = 2) or severe hypertension (*n* = 1). MAP and CPP was available for 4 stroke and 3 sham animals due to arterial line failure in other animals. There were no significant differences in the physiological parameters of heart rate, mean arterial pressure or cerebral perfusion pressure within or between groups at any time (*p* > 0.05; Table 1).

**Table 1:** Physiological parameters of sham and stroke mice (*n* = 6/group).

Parameter	Sham	Stroke
HR (BPM)		
1.0 h	282.5 ± 38.5	240.1 ± 39.7
23.5 h	251.8 ± 55.7	255.5 ± 26.0
24.0 h	260.6 ± 45.5	254.5 ± 35.0
MAP (mmHg)		
23.5 h	74.3 ± 9.2	78.4 ± 5.2
24.0 h	75.0 ± 8.1	75.2 ± 3.6
CPP (mmHg)		
23.5 h	69.8 ± 9.5	67.6 ± 4.6
24.0 h	70.4 ± 8.1	62.8 ± 5.0

*Note*: Within group values were compared using paired *t*‐tests. Between group comparisons were made using Student's *t*‐test. all *p* > 0.05.

Abbreviations: BPM, beats per minute; CPP, cerebral perfusion pressure; h, hour(s); HR, heart rate; MAP, mean arterial pressure.

**Figure 1:**
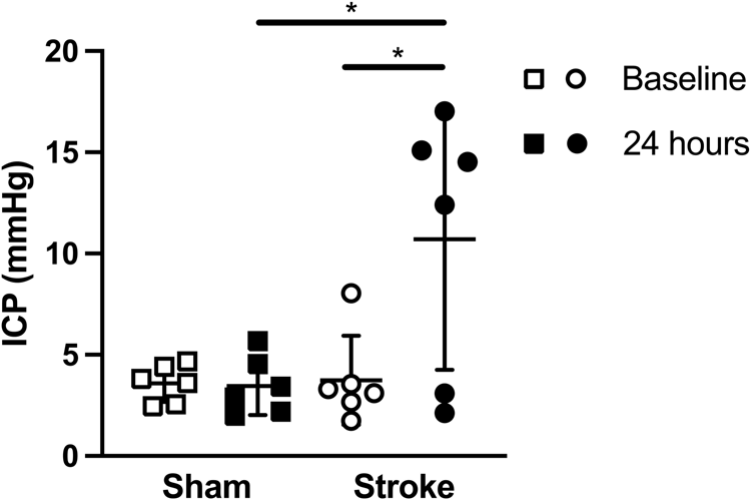
Intracranial pressure (ICP) increases in mice after photothrombotic stroke (*n* = 6/group). ICP at 24 h post‐stroke was significantly elevated compared to baseline (1 h post‐stroke) and sham at 24 h. *p < 0.05. Data presented as mean ± standard deviation.

Baseline ICP was not significantly different between groups (stroke: 3.7 ± 2.2 mmHg; sham: 3.6 ± 0.9 mmHg; *p* = 0.9; Figure 1). From baseline, ICP increased in 4 of the 6 stroke mice. The group average for all 6 stroke animals showed a significant increase to 8.9 ± 5.3 mmHg at 23.5 h (*p* = 0.03) and 10.7 ± 6.5 mmHg at 24 h (*p* = 0.03; Figure 1). This was also significantly higher than ICP in sham animals at 23.5 h (3.5 ± 1.3 mmHg; *p* = 0.04) and 24 h (3.45 ± 1.43 mmHg; *p* = 0.02; Figure 1). There was no change in ICP between baseline and 23.5 or 24 h in sham animals (*p* = 0.9; Figure 1).

Infarct volume corrected for edema in stroke animals was 15.9 ± 9.0 mm^3^, representing 9.7 ± 5.0% of the ipsilateral hemisphere. Edema volume was extremely small and not significantly different between stroke and sham animals (0.04 ± 0.04 mm^3^ vs. 0.01 ± 0.01 mm^3^, *p* = 0.2). The two stroke mice with no ICP rise were seen to have minimal infarcted tissue.

## 4 DISCUSSION

In this study, we have shown that an increase in ICP at 24 h post‐stroke, seen by us and others in rats^3^, ^4^, ^5^, ^10^ and sheep^8^ using large vessel occlusion models, is also seen in mice. This ICP rise occurred in the presence of small infarct volumes (~10% of the ipsilateral hemisphere volume) with little or no edema. This data presents additional evidence for alternative mechanisms of ICP elevation post‐stroke. Infarct volumes were spontaneously dichotomous which may reflect natural variation in stroke induction, although the photothrombotic stroke model typically has less variability than other models, such as middle cerebral artery occlusion. We believe this variation is acceptable given the variation in human stroke. There was a 2.4‐fold average rise in ICP post‐stroke, even including the two animals with minimal infarction and no ICP rise. Edema volumes were negligible in all animals, excluding this as the cause of ICP rise.

Evidence for large stroke as a sole cause of ICP elevation remains controversial. In fact, clinical studies have demonstrated that patients with malignant infarcts can experience herniation despite normal ICP.^22^, ^23^ Studies in rats have reported an increase in CSF outflow resistance after stroke, suggesting that an increase in CSF volume is a potential contributor to our observed ICP elevation.^6^, ^9^ However, studying total CSF volume is technically challenging. We have recently shown a significant reduction in CSF flow within the parenchyma and outflow to the nasal mucosa at 24 h in the same mouse model of stroke,^19^ highlighting the potential importance of the CSF system in stroke sequalae. If CSF volume is indeed a potential contributing factor to the ICP rise reported in this and previous rodent studies, targeting CSF dynamics, specifically production or outflow, may be a potential new target for countering post‐stroke ICP elevation.

The importance of these findings is not just limited to mechanisms of ICP elevation. Our work in rats also shows a potential therapy for completely preventing this ICP rise—short duration hypothermia.^3^, ^4^, ^5^, ^24^ Given the similarities we have observed across species with ICP rise, it would be interesting to see if hypothermia has similar effects in mice and higher order animals.^25^

There are few studies of ICP in mice, potentially due to the complexity of monitoring ICP in this species given the large size of available pressure probes compared to the small mouse brain. We used epidural monitoring in this study, as mice have a very thin and delicate dura which does not create a barrier to ICP measurement, unlike in humans.^26^ Epidural monitoring is also more appropriate as inserting a probe into tissue or the lateral ventricle, as is the gold standard in humans, has the potential to cause significant tissue damage in rodents, especially over time,^27^ and confound changes in ICP related to an induced injury. Monitoring of ICP from more invasive locations is more feasible in gyrencephalic animals given larger brain volumes. However, similar ICP elevations as reported here have not been observed in species other than sheep due to a lack of monitoring after 18 h post‐stroke.^28^, ^29^, ^30^, ^31^

A potential limitation of this study is the lack of a pre‐stroke ICP baseline. It was not possible to measure ICP before stroke induction as the screw and probe for ICP monitoring would have obstructed placement of the light to induce thrombosis. However, we do not believe this to have altered the results given that stroke and sham mice had comparable baseline ICPs measured at 1 h post‐stroke/sham induction.

In conclusion, this study indicates that ICP more than doubles after small strokes in mice. The mechanism remains unclear but does not appear to be related to cerebral edema. These findings indicate that the current understanding of post‐stroke ICP elevation is incomplete and new strategies may be required to protect the brain following ischemic stroke.

## AUTHOR CONTRIBUTIONS

Kirby E. Warren and Neil J. Spratt developed the study. Kirby E. Warren completed all surgical and tissue processing procedures, with assistance from Rebecca J. Hood, Kirby E. Warren, and Rebecca J. Hood performed statistical analysis. Kirby E. Warren and Rebecca J. Hood wrote the manuscript. Fredrick R. Walker provided guidance and laboratory space.

## CONFLICT OF INTEREST STATEMENT

The authors declare no conflict of interest.

## ETHICS STATEMENT

The study protocol was approved by the Animal Care and Ethics Committee of the University of Newcastle (protocol code A‐2013‐340).

## Supporting information

Supporting information.

## Data Availability

The data presented in this study is available in the article/supplementary materials.
