# Supplementary material for: Intracranial pressure is elevated at 24 h post‐stroke in mice
Source: Neuroprotection. 2024 Feb 25;2(1):60–4. doi: 10.1002/nep3.36 (PMC12486897; doi:10.1002/nep3.36)

Supplementary figure 1. Representative image of 2,3,5-Triphenyl tetrazolium chloride-stained brain sections. A. stroke, B. sham


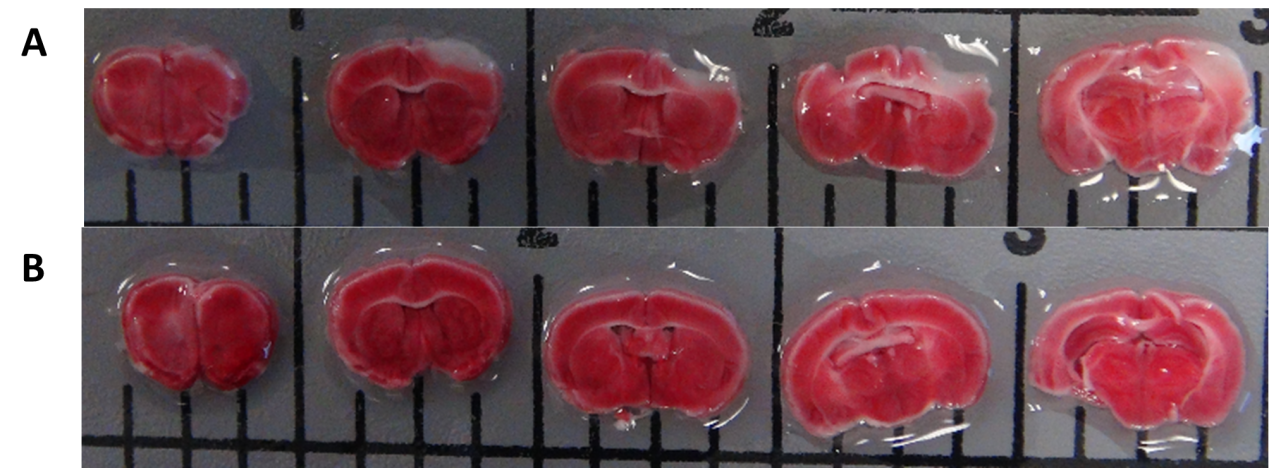

Supplement: Supplementary file 1 — Supporting information. [file NEP3-2-60-s001.docx]
